# Predictive Modeling and Experimental Optimization of the Electrodeposition–Sintering Process for Functional Ceramic Coatings

**DOI:** 10.3390/ma18163893

**Published:** 2025-08-20

**Authors:** Jesús M. Rodríguez-Rego, Antonio Macías-García, Laura Mendoza-Cerezo, Juan Pablo Carrasco-Amador, Antonio Díaz-Parralejo

**Affiliations:** 1 Departamento de Expresión Gráfica, Universidad de Extremadura, Avenida de Elvas, s/n, 06006 Badajoz, Spain; jesusrodriguezrego@unex.es; 2 Departamento de Ingeniería Mecánica, Energética y de los Materiales, Universidad de Extremadura, Avenida de Elvas, s/n, 06006 Badajoz, Spainadp@unex.es (A.D.-P.); 3 Departamento de Bioquímica, Facultad de Ciencias, Escuela de Ingenierías Industriales, Universidad de Extremadura, Avenida de Elvas, s/n, 06006 Badajoz, Spain

**Keywords:** design of experiments (DoE), electrodeposition, engineering visualization, material characterization

## Abstract

This study focuses on optimizing a sol–gel based electrodeposition–sintering process for producing yttria-stabilized zirconia (YSZ) ceramic coatings on stainless steel substrates. Four key process variables—precursor concentration, current density, sintering time, and temperature—were evaluated in terms of two response variables: R (electrodeposition yield) and S (sintering yield). A fractional factorial design was used to reduce the number of experiments while enabling robust statistical modeling. Multiple linear regression analysis revealed that precursor concentration and current density were the most influential factors for both R and S, whereas sintering time and temperature had a lesser effect. Under central conditions (42.9 g·L^−1^, 1.5 A·cm^2^, 500 °C, 20 min), coatings exhibited yields of ~3.9 mg·cm^2^ and superior morphological uniformity. Higher current density (3 A·cm^2^) increased R to 6.9 mg·cm^2^ but induced porosity and cracking. Compared to conventional sol–gel derived coatings, the proposed methodology enables a more controlled microstructure with a trade-off between mass deposition and structural integrity. This predictive, statistically validated approach facilitates the optimization of electrodeposition parameters to obtain defect-minimized ceramic coatings, particularly suited for protective and thermal barrier applications in demanding environments.

## 1. Introduction

The current industry demands advanced materials with high corrosion resistance, particularly for critical applications in the biomedical, aerospace, and energy sectors [1,2]. Among these, stainless steels are widely used; however, in extreme environments, additional protective coatings are often required [3,4]. Various materials, such as nanocomposites, bimetallic metals, and yttria-stabilized zirconia (YSZ) coatings, offer effective solutions, each with distinct advantages in terms of efficiency and durability [5,6,7].

Ceramic materials, particularly yttria-stabilized zirconia (YSZ, ZrO_2_–Y_2_O_3_), are widely applied due to their excellent thermal resistance, chemical stability in aggressive environments, and mechanical properties [8,9,10,11]. These coatings can be deposited using various techniques such as CVD, PVD, or sol–gel methods [12,13,14,15]. The sol–gel method is a cost-effective and efficient technique for depositing thin films, offering significant advantages in terms of cost, uniformity, and application flexibility. While CVD and PVD each have their own strengths, the sol–gel process stands out for its simplicity and capability to uniformly coat large surface areas.

The electrodeposition of YSZ coatings from sol–gel solutions is a promising technique for application to metallic materials. This method allows precise control over the stoichiometry and morphology of the deposited layers, which is critical for enhancing the performance and stability of these materials [16,17]. However, recent studies have shown that the quality of the coating is strongly influenced by precursor concentration, current density, and deposition time [18,19,20,21]. These parameters must be jointly optimized to achieve high-quality coatings with desirable properties such as corrosion resistance and uniformity. These properties include low porosity, strong adhesion, uniform thickness, and structural integrity. High current density may increase deposition rate but often leads to internal stress and cracking, while precursor concentration directly affects coating density and homogeneity.

This study addresses this challenge through the application of the Design of Experiments (DoE) methodology, which enables the evaluation of the influence of these variables on coating formation with a minimal number of tests [22,23,24]. Such methodologies allow for more precise and predictable control of the coating process, ensuring the attainment of desired properties with reduced experimental workload. The results obtained may have direct implications in various fields such as medical implants (biocompatible coatings), energy (thermal barrier coatings in turbine blades), and electronics (gas sensors), among others.

Therefore, the objective of this study is to optimize the electrodeposition–sintering process parameters for the fabrication of YSZ ceramic coatings on stainless steel substrates, aiming to maximize the deposition efficiency while ensuring coating quality and uniformity. To achieve this, a fractional factorial Design of Experiments (DoE) was implemented to systematically evaluate the influence of precursor concentration, current density, sintering time, and temperature on two response variables: electrodeposition yield (R) and sintering yield (S). The methodology combines statistical modeling, experimental testing, and optical microscopy to obtain predictive equations and assess the structural quality of the resulting coatings.

The results confirm that the proposed model successfully identifies optimal processing conditions that maximize coating yield while minimizing structural defects, providing a validated approach for controlled and reproducible ceramic coating fabrication.

## 2. Materials and Methods

### 2.1. Starting Materials

The substrates used are made of AISI 310 (P) stainless steel. Table 1 shows the data relating to the chemical composition and dimensions of the test pieces (P).

The AISI 310 (P) stainless steel test tubes are embedded in TransOptic resin, supplied by Buehler, in order to facilitate their handling and the mechanical treatments to which they will be subjected. The embedding was carried out using a Buehler Simplimet 1000 automatic mounting press (Buehler, Lake Bluff, IL, USA), following the parameters specified in Table 2.

Once the tableting process was complete, the substrates were sanded and polished. A Buehler Phoenix 4000 (Buehler, Lake Bluff, IL, USA) polisher was used for this purpose.

Test piece P was sanded using silicon carbide (SiC) discs, gradually increasing the grain size from 240 to 400 and finally to 800 grains, with times of 5, 8, and 10 min, respectively. Distilled water was used as a lubricant.

Subsequently, polishing was carried out in three phases with a monocrystalline suspension of Buehler diamond particles. In each phase, diamond paste with particle sizes of 9, 6, and 3 microns was used, applied for 15 min at each stage. These mechanical treatments resulted in the test pieces designated PM.

The test pieces were then unmolded.

Subsequently, before applying the coating, some PM test pieces were subjected to a chemical oxidation treatment in a controlled atmosphere. This process was carried out in an oven at 300 °C for 2 h, with the aim of generating a surface oxide layer that would promote the adhesion of the coating. As a result of these treatments, the PMOx test pieces were obtained.

#### Mechanical Characterization of the Starting Materials

AISI 310 stainless steel was characterized using a Dataview TR-200 roughness tester (manufactured by Beijing TIME High Technology Ltd., headquartered in Beijing, China). The surface roughness parameters Ra, Ry, and Rz were recorded to assess the effect of the mechanical and chemical treatments.

### 2.2. Preparation of the Solution

The alcoholic solution of zirconium alkoxide was obtained by mixing and subsequently stirring the precursor zirconium n-propoxide (ZNP), composed of 70 wt% n-propanol, with 1-propanol (PrOH), using nitric acid (HNO_3_) as a catalyst. The molar ratio of the components was ZNP/PrOH/H_2_O/HNO_3_ = 1/15/6/1.

To prepare the precursor solution intended for ZrO_2_ coatings with 3 mol% Y_2_O_3_, the initial solution was mixed with a solution of yttrium acetate (AcY·4H_2_O) dissolved in isopropyl alcohol (2-PrOH), which had been previously acidified with nitric acid. The molar ratio of the components in this mixture was AcY/2-PrOH/H_2_O/HNO_3_ = 1/15/6/1, and the oxide concentration of the stock solution reached 78 g·L^−1^.

To investigate the effect of oxide concentration in this study, the stock solution (78 g·L^−1^) was diluted with n-propanol to obtain oxide concentrations of 60.45 g·L^−1^, 42.9 g·L^−1^, 25.35 g·L^−1^, and 7.8 g·L^−1^.

#### Characterization of Solutions

The solutions were characterized by measuring density, viscosity, and pH at room temperature [3,26,27]. Densities (ρ) were determined using a 10 mL Lussac pycnometer (accuracy ± 0.1), and masses were measured with a Sartorius analytical balance, model BP 121S, with a deviation of 0.1 mg. Viscosity (η) was calculated using an Ostwald viscometer. Finally, the pH of the solutions was measured with a Hanna pH meter, model HI 3220.

### 2.3. Design and 3D Printing of the Electrodeposition Cell

Using parametric design software and taking advantage of rapid prototyping through 3D printing, an electrodeposition cell adapted to the specific requirements of the substrate and solution was developed. To do this, Autodesk Inventor^®^ parametric drawing software (Inventor: 2024) was used to convert the initial designs into three-dimensional models. Subsequently, UltiMaker Cura^®^ software (Version 5.9.1) was used to generate the necessary code, which was interpreted by a fused deposition 3D printer to produce the designed electrodeposition cell.

**Figure 1:**
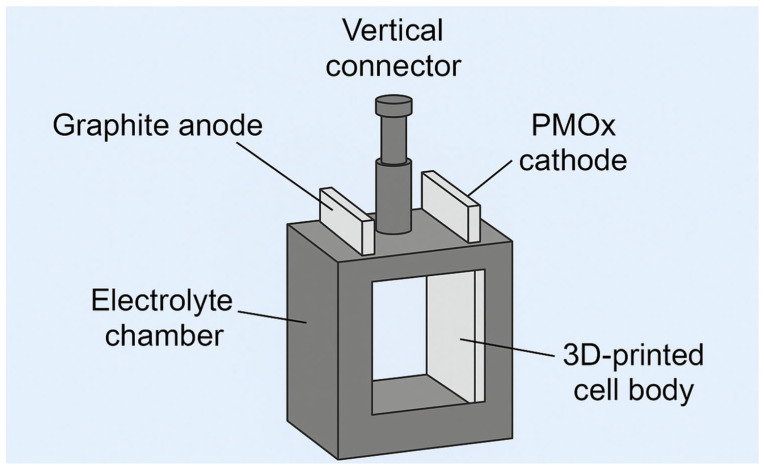
Schematic design of the custom-built 3D-printed electrodeposition cell. The system includes a graphite anode (**left**), a PMOx cathode (**right**), and a central electrolyte chamber. The entire structure is supported by a rigid 3D-printed frame, and the vertical connector ensures electrical contact and mechanical stability during deposition (Own source).

The manufacture of the cell with an optimal three-dimensional design has enabled tests to be carried out that would otherwise have been limited to commercially available solutions (Figure 1).

### 2.4. Design of Experiments (DoE)

The detailed study of complex physicochemical processes, such as electrodeposited coatings, requires a rigorous methodology that enables the identification and quantification of the influence of multiple variables on the desired response. In this context, Design of Experiments (DoE) provides a valuable approach for obtaining relevant information on process behavior.

In this study, an experimental design was employed to analyze the influence of multiple factors on the electrodeposition and sintering processes. The main objective was to evaluate and quantify the impact of the independent variables on the response variables. The independent variables considered were precursor concentration (g/L), current density (A/cm^2^), time (min), and sintering temperature (°C), while the response variables were the electrodeposition yield (R) and the sintering yield (S).

The experimental design included 36 trials, covering all possible combinations of the levels of the independent variables. From this design, coded tables were generated with the 36 experimental runs, along with decoded tables that included the values obtained for R and S. These data were analyzed using statistical techniques, including descriptive statistics and regression analysis.

The descriptive analysis summarized the main features of the response variables (R and S), providing initial information on their distribution and variability. Parameters such as mean, standard deviation, minimum, and maximum values were calculated. Additionally, an analysis of variance (ANOVA) was performed to assess the significance of the independent variables and their interactions.

Multiple regression analysis was used to model and quantify the relationships between the independent variables (concentration, current density, time, and sintering temperature) and the response variables (R and S). This predictive model provided insight into how variations in the independent variables affect the outcomes of the process.

### 2.5. Electrodeposition Coating and Sintering

Initially, the dip-coating technique was employed [28,29] to evaluate the withdrawal speed, and the results were analyzed accordingly. After testing various speeds, a withdrawal rate of 10 mm·min^−1^ was selected for the samples. Based on these preliminary findings, the coating process was subsequently carried out on PMOx samples using the electrodeposition technique (ELD) [30,31]. In this process, PMOx samples served as the cathode, graphite electrodes were used as the anode, and the solution was continuously stirred to ensure homogeneity and prevent particle sedimentation.

Electrodeposition is an electrochemical process in which, upon application of an electric current, the coating material present in the solution precipitates onto the substrate (cathode), forming a coating layer. This procedure was carried out using a modified KSV NIMA system controlled by dedicated software. The power supply used was a BIO-RAD unit (Model: PowerPac HV Power Supply), operating within a voltage range of 100–120/220–240 V.

Process conditions included solution concentrations ranging from 7.8 to 78 g·L^−1^, sintering temperatures from 200 to 800 °C, current densities between 0 and 3 A·cm^−2^, and substrate residence times in the electrodeposition bath between 2 and 38 min.

After electrodeposition, the samples underwent thermal treatment (sintering) in a Lenton furnace operating under atmospheric conditions. A controlled heating and cooling rate of 10 °C·min^−1^ was applied to minimize thermal stresses, in accordance with standard practices for ceramic materials. Initially, the samples were dehydrated by heating to 100 °C and holding for 60 min. Subsequently, sintering was performed in the same furnace at 200, 350, 500, 650, and 800 °C for 120 min.

#### Characterization of Sintered Coatings

The samples were characterized using optical microscopy to assess the quality of the coatings applied to the substrates and detect the possible presence of cracks or fissures. For this analysis, a Nikon Epiphot 300 reflected light microscope equipped with an integrated photography system was used.

## 3. Results and Discussion

### 3.1. Characterization of the Test Specimens

In this study, AISI 310 (P) stainless steel test tubes were used as initial substrates. These test tubes were first subjected to mechanical treatment (PM) and then to chemical treatment (PMOx).

The test tubes obtained were characterized using roughness measurement and optical microscopy in order to evaluate the influence of the surface condition on the coating processes.

Table 3 presents the average surface roughness values for the different samples studied: P, PM, and PMOx. Upon analyzing the results, clear trends can be observed that reflect the effects of the mechanical and chemical treatments applied. The initial sample, P, shows Ra (0.106 μm) and Rz (0.930 μm) values slightly higher than those reported in the literature for similar stainless steels [32]. This may be attributed to various handling processes the sample underwent, such as transport, washing, and drying. These values indicate that the surface of sample P is not entirely smooth and may be deteriorated, which could negatively affect the uniformity of subsequent coatings.

The PM sample, after mechanical treatment (grinding and polishing), exhibits a notable reduction in roughness values for all parameters analyzed (Ra = 0.090 μm, Rz = 0.389 μm). This decrease demonstrates the effectiveness of the treatment in eliminating surface defects present in the original sample P, resulting in a more uniform surface, better suited for the following coating stages.

Finally, when comparing the PM and PMOx samples, the roughness values of PMOx are slightly higher (Ra = 0.098 μm, Rz = 0.477 μm), suggesting that the deposition of the oxide layer causes a slight increase in roughness. However, in comparison to the original sample P, the oxidized surface is more homogeneous, which may enhance the adhesion of subsequent coatings. These findings are consistent with the literature [4], which indicates that chemical treatment induces minor changes in roughness without compromising surface uniformity. These results show that the applied mechanical and chemical treatments significantly improve the surface quality of the samples. The reduction of initial roughness in the PM sample and the slight added homogeneity in PMOx are key factors for optimizing coating processes.

**Figure 2:**
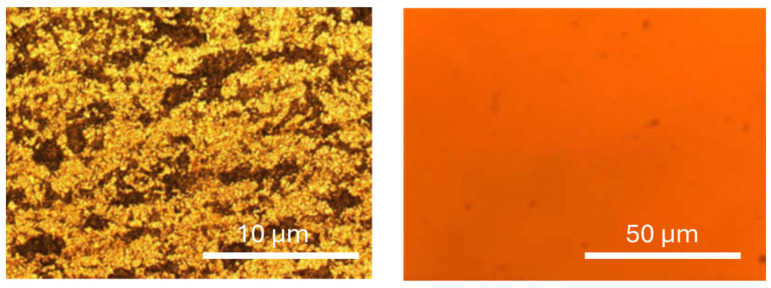
Optical microscopy images of the mechanically polished stainless steel sample (PM, **left**) and the oxidized sample (PMOx, **right**). Different magnifications were selected to emphasize the specific surface features of each sample. Bright areas correspond to smoother and more reflective regions, while dark areas indicate surface roughness, defects, or microstructural irregularities [25].

In addition, Figure 2 presents the optical micrographs corresponding to some of the samples analyzed in this study.

The comparative analysis of the images obtained for samples PM and PMOx, shown in Figure 2, reveals differences in surface uniformity and topography. The PM sample (left) was observed at higher magnification to highlight the microtexture resulting from mechanical polishing, where bright areas represent smoother, more reflective regions, and dark zones correspond to surface defects or irregularities. In contrast, the PMOx sample (right) was imaged at lower magnification to capture the overall homogeneity of the oxide layer formed after thermal treatment.

At 300 °C in ambient air, AISI 310 stainless steel is expected to form a thin, uniform oxide layer, primarily composed of chromium-rich oxides (e.g., Cr_2_O_3_), which act as protective barriers. These oxides typically appear as smooth, continuous films with nanometric to submicrometric thickness. This is consistent with the PMOx surface morphology observed in the micrograph, which shows a homogeneous orange layer and fewer visible defects compared to the untreated sample. Despite the difference in scale, the images support the roughness measurements and indicate that oxidation leads to a more uniform and defect-free surface.

Thus, the mechanical treatment followed by oxidation proves effective in improving the surface characteristics of the initial P test piece, resulting in a more homogeneous and oxidation-stabilized surface [4].

### 3.2. Characterization of the Standard Solution

The ZrO_2_-3mol% Y_2_O_3_ solutions were characterized by measuring their pH, density, and viscosity at a temperature of 22 °C. The results obtained showed a pH of 0.5, a density of 0.966 g·cm^−3^, and a viscosity of 0.8904 cP. These values correspond to a molar ratio of the components (ZNP-AcY)/alcohol/water/nitric acid of 1/15/6/1, which is equivalent to an oxide concentration of 78 g·L^−1^.

The characterized parameters reflect significant properties of the standard solution. The acidic pH (0.5) may play a key role in the stability of the solution and in the interaction mechanisms between the components, ensuring adequate dispersion of ZrO_2_ and Y_2_O_3_. In addition, the low density (0.966 g·cm^−3^) and viscosity (0.8904 cP) are indicators of a fluid solution, which is essential to facilitate processes such as electrodeposition or the uniform formation of coatings.

The inclusion of Y_2_O_3_ in the preparation of ZrO_2_ solutions has the main objective of improving the corrosion resistance of the ceramic coating. This improvement is based on yttrium’s high affinity for oxygen, preventing oxygen atoms from penetrating the metal substrate. Y^3+^ ions act as “capturers” of oxygen atoms on the surface of the coating, thus reinforcing its protective capacity [33].

### 3.3. Design of Experiments (DoE)

The objective of applying DoE is to significantly reduce experimental costs and times and analyze the interactions between the independent variables and their influence on the coating process. This approach represents an efficient strategy for improving complex industrial processes.

A full factorial design (3^4^ = 81) involves a large number of trials. To reduce this number, a 3^4^ − 1 fractional factorial design was used, which reduced the number of experiments to 36. This design focused on optimizing the electrodeposition (ELD) coating process of PMOx test tubes, evaluating the influence of four independent variables: solution concentration, current density, sintering time, and temperature.

The tests will be distributed as follows: 27 tests of the 3^4^ − 1 fractional design, for the selection of key combinations, 6 central tests to evaluate quadratic effects and curvature, and 3 randomly replicated tests to estimate experimental error.

The four independent variables selected for this study are each defined by their minimum and maximum values, a central value, and a step, as shown in Table 4.

The use of a fractional factorial design combined with replicated central points provides an appropriate balance between reducing the number of experiments and maintaining statistical analysis quality. The central values (concentration: 42.9 g/L, current density: 1.5 A/cm^2^, time: 20 min, and sintering temperature: 500 °C) serve as reference points, enabling comparisons of variations in the independent variables and their impact on the response variables. This approach enhances the accuracy of data interpretation and facilitates the identification of trends and nonlinear relationships.

The response variables, R (electrodeposition yield) and S (sintering yield), allow for direct evaluation of the properties of the resulting material. Furthermore, the fractional design ensures the identification of critical parameter combinations, while the central and replicated trials contribute to statistical robustness, allowing for the estimation of quadratic effects and the minimization of error.

Through the implementation of the experimental design, coded (Table 5) and decoded (Table 6) matrices were generated to organize the data and facilitate subsequent analysis. These tables, together with the experimental results, provide a solid foundation for optimizing process performance.

The decoding of Table 5 was performed using the inverse of the coding formula:

Real value = Coded value × Step + Central value

Based on the reference values in Table 4:

Concentration: Central = 42.9, Step = 17.55

Temperature: Central = 500, Step = 150

Current density: Central = 1.5, Step = 0.75

Time: Central = 20, Step = 9

By applying this formula to each coded value listed in Table 5, the decoded Table 6 is obtained.

### 3.4. Coating Process Performance

The statistical design of experiments (DoE) described above aims to optimize the electrodeposition coating (ELD) process on steel test pieces. To this end, four independent variables were used: solution concentration, current density, electrodeposition time, and sintering temperature. Based on the experimental combinations described in Table 6, the coatings were applied to each sample, generating the following responses: electrodeposition yield (R) and sintering yield (S), which indicate the amount of material deposited and sintered per unit area, respectively. The results obtained are shown in Table 7.

In view of the above results, it can be seen that concentration and current density appear to be the variables that most influence process performance. Likewise, the central conditions provide stable but not optimal performance, suggesting that extreme conditions (high concentrations, temperatures, and current densities) are necessary to maximize performance.

The sample values, S < R, indicate that there is a loss of material during sintering. Thus, the electrodeposition yield ranges from 0.85 mg/cm^2^ (sample 26) to 6.90 mg/cm^2^ (sample 20), while the sintering yield varies from 0.70 mg/cm^2^ (sample 26) to 5.79 mg/cm^2^ (sample 20). These losses could be due to evaporation of volatile components during sintering, formation of pores or cracks due to thermal contraction, or chemical reactions (e.g., oxidation or decomposition of doped zirconia).

In view of the design of experiment (DoE), it can be seen that the experimental conditions have a considerable impact on the performance of the process. Thus, the central tests (samples 1, 3, 4, 5, 6, 10, 25, 27, 28) show consistent yields (R ≈ 3.88 mg/cm^2^, S ≈ 3.30 mg/cm^2^), suggesting that the central conditions provide stable performance. The replicated samples (e.g., samples 1, 3, 4, 5, and 6) show very similar yields, indicating good reproducibility of the process under the same conditions. Likewise, high yields correspond to high values of the independent variables. Thus, samples with higher concentrations (e.g., sample 15 with 78 g/L) tend to have higher yields (R = 5. 97 mg/cm^2^, S = 4.68 mg/cm^2^), samples with higher current density (e.g., sample 20 with 3 A/cm^2^) have the highest yields (R = 6.90 mg/cm^2^, S = 5.79 mg/cm^2^), and electrodeposition time also influences yield. This variability indicates that the experimental conditions have a considerable impact on the performance of the process.

In order to further investigate the results obtained with the coatings, a statistical analysis was carried out.

### 3.5. Statistical Analysis

Statistical analysis was performed on the R and S values collected in Table 7. The descriptive statistics values for R and S are shown in Table 8.

Based on Table 8, it can be observed that the R values have a mean of 3.78 and a standard deviation of 1.34, indicating a moderate dispersion around the mean. Likewise, the range of 6.05 suggests a significant variability in the results depending on the experimental conditions.

In contrast, the S values show a mean of 3.24 and a standard deviation of 1.23, indicating a slightly lower dispersion compared to R. In this case, the range of 5.09 also reflects variability, although less pronounced than in R.

In summary, the analysis of all data provides a more comprehensive understanding of the variability and behavior of R and S in the experiment. It reveals that R is more variable than S, and that both responses are significantly influenced by the independent variables.

### 3.6. Regression Analysis

A multiple linear regression model was selected due to its ability to relate multiple independent variables and assess their effects simultaneously. This multiple linear regression approach enables the correlation of the independent variables (C, T, DC, t) with the dependent variables (R and S).

**Figure 3:**
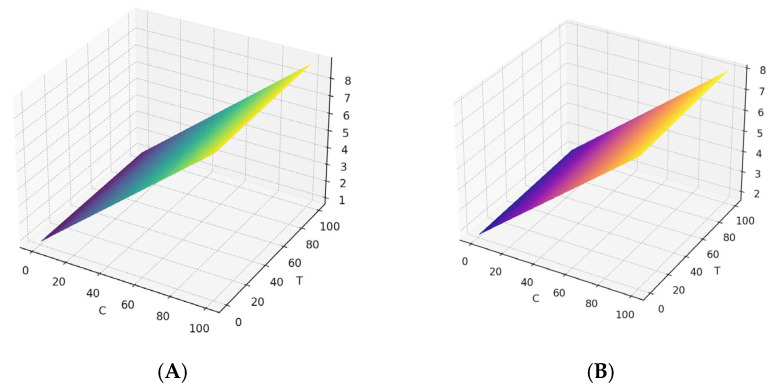
(**A**) Regression curve: R versus C and T (DC = 1) and (**B**) regression curve: S versus C and T (DC = 1, t = 50).

Model fitting was carried out using the least squares method, which minimizes the sum of the squared errors between the observed values and those predicted by the model. Separate regression models were fitted for each response variable (R and S). The resulting regression equations for R and S are presented in Table 9 and Figure 3.

To assess the quality of the model fitting, an analysis was performed on the regression coefficients, the coefficient of determination (R^2^), and the *p*-values.

The regression coefficients indicate the magnitude and direction of the relationship between each independent variable and the response variable. The interpretation of each term in the regression equations for R and S is provided below. The constant term (−0.45 for R and −0.35 for S) represents the expected value of the response variable when all independent variables are set to zero. The coefficients for concentration (0.08 for R and 0.06 for S), temperature (0.002 for R and 0.003 for S), current density (1.20 for R and 1.05 for S), and time (0.03 for R and 0.02 for S) indicate the change in the response variable per unit increase in each independent variable, assuming the others remain constant. A positive coefficient suggests that an increase in the corresponding independent variable tends to increase the values of R and S, although to a relatively small extent.

The results obtained through the regression equations reveal that concentration and current density exert the most significant influence on the response variables R and S. While concentration positively affects both R and S, its influence is less pronounced than that of current density.

In conclusion, these regression equations not only allow for the prediction of R and S values based on the independent variables but also provide a quantitative tool for evaluating the individual contribution of each independent variable to the observed responses.

The R^2^ value measures the proportion of the variability in the response variable that is explained by the model. As shown in Table 9, the coefficient of determination R^2^ = 0.92 indicates that 92% of the variability in R is explained by the model, while R^2^ = 0.89 accounts for 89% of the variability in S.

Furthermore, the *p*-values assess the statistical significance of the regression coefficients for R and S. These values are presented in Table 10.

Based on the table above, it can be stated that if *p* < 0.05, the variable is considered statistically significant for the model. Therefore, in the case of R, temperature (T) is not significant (*p* > 0.05), suggesting it does not have a statistically meaningful impact on R. This is consistent with the experimental context, as temperature does not play a role in the electrodeposition process but is relevant during sintering. However, for S, all variables are statistically significant (*p* < 0.05).

In summary, the regression models for R and S are highly predictive, as the R^2^ values are close to 1. Furthermore, to improve the R model, it may be appropriate to remove the variable T (temperature), given its lack of significance. In contrast, for the S model, all variables are relevant and should be retained.

In addition, the regression equations enabled the identification of optimal processing conditions. The combination of 42.9 g·L^−1^ precursor concentration, 1.5 A·cm^2^ current density, 20 min of deposition time, and 500 °C sintering temperature was found to produce coatings with the highest yield and best uniformity. These conditions were validated through independent experimental replicates, confirming the accuracy and predictive value of the proposed model.

### 3.7. Characterization of the Coatings by Optical Microscopy

To differentiate between the amount of material deposited on the specimens and the quality of the coating after sintering, optical microscopy characterization was carried out. For this purpose, the most significant variables, namely, current density and concentration, were analyzed.

Optical micrographs were obtained using 5× and 10× objective lenses. Figure 3 presents the optical micrographs of sample 1 (coded as 0,0,0,0; decoded: 42.9 g·L^−1^, 500 °C, 1.5 A·cm^−2^, 20 min) and sample 20 (coded as 0,0,2,0; decoded: 42.9 g·L^−1^, 500 °C, 3 A·cm^−2^, 20 min), allowing for the analysis of the influence of current density while keeping concentration, temperature, and residence time in the custom-designed electrolytic cell constant.

Sample 20 exhibited an electrodeposition yield of 6.90 mg·cm^−2^ and a sintered yield of 5.79 mg·cm^−2^, whereas sample 1 showed an electrodeposition yield of 3.89 mg·cm^−2^ and a sintered yield of 3.30 mg·cm^−2^.

Samples 1 and 20 were selected to evaluate the influence of current density on the electrodeposition process, while maintaining constant precursor concentration (42.9 g·L^−1^), sintering temperature (500 °C), and residence time (20 min). This allows the effect of current density to be assessed in isolation.

Based on the experimental results and optical micrographs, it can be observed that doubling the current density from 1.5 A·cm^−2^ (sample 1) to 3 A·cm^−2^ (sample 20) leads to a significant increase in electrodeposition yield (~77%). This is attributed to the fact that higher current density promotes a greater amount of material deposition. Sintering losses are comparable in both cases (15.2% and 16.1%, respectively), suggesting that the loss mechanism is not strongly dependent on the current density and is likely associated with volatilization, surface oxidation, or densification shrinkage during sintering [34,35,36].

**Figure 4:**
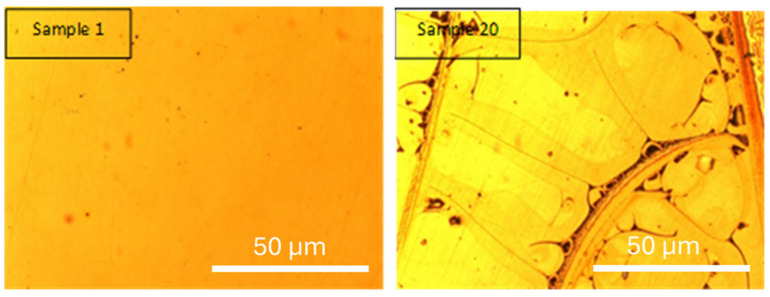
Optical microscopy of samples 1 and 20 [25].

Figure 4 shows the optical micrographs of sample 1 (central condition) and sample 20 (extreme condition). Sample 1 reveals a uniform and homogeneous coating, attributed to the moderate deposition rate, which allows a gradual, stable film growth with fewer structural defects. In contrast, sample 20 exhibits a more irregular and textured morphology, with dark lines and heterogeneous areas. These features suggest that high current density may induce localized stress, non-uniform nucleation, and increased brittleness due to rapid material accumulation.

In both cases, the deposited film corresponds to a yttria-stabilized zirconia (YSZ) layer formed via sol–gel electrodeposition. Although no phase-specific analysis was conducted at this stage, the observed morphologies are consistent with thin ceramic films where process parameters critically affect microstructure. The comparison between these samples highlights the central role of current density in determining the coating’s integrity and performance.

**Figure 5:**
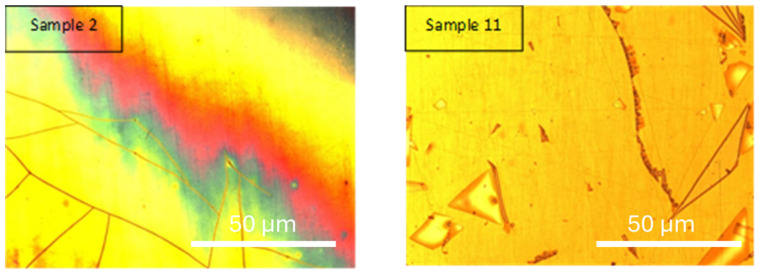
Optical microscopy of samples 2 and 11 [25].

Samples 2 and 11 (Figure 5) were selected to evaluate the effect of precursor concentration on the morphology and performance of the electrodeposited coatings. Both samples were processed under identical conditions of sintering temperature (650 °C), current density (2.25 A·cm^2^), and deposition time (29 min), which allows the variable “concentration” to be isolated and studied independently.

Sample 2, with a higher precursor concentration (60.45 g·L^−1^), achieved an electrodeposition yield of 6.80 mg·cm^−2^ and a sintered yield of 5.36 mg·cm^−2^. In contrast, sample 11, with a lower concentration (25.3 g·L^−1^), yielded 4.08 mg·cm^−2^ and 3.12 mg·cm^−2^, respectively. These differences confirm that concentration is a key factor in determining deposition rate and overall yield.

Both samples exhibit surface domains with angular geometries and visible lines separating regions of different contrast. However, sample 11 shows more pronounced and well-defined domains, along with finer crack patterns, suggesting a less cohesive and potentially brittle structure. These features may be attributed to slower nucleation kinetics and greater localized stress formation at lower concentrations. The observed triangular structures may be associated with differential grain growth, while the cracks could result from shrinkage during drying or from internal stress developed during sintering.

In contrast, sample 2 exhibits a smoother and more continuous morphology, with fewer visible defects. The higher concentration may promote more uniform nucleation and faster particle aggregation, leading to denser coatings with better structural integrity.

Although no specific phase analysis was performed in this section, both samples are expected to contain thin films of yttria-stabilized zirconia (YSZ), formed through sol–gel electrodeposition. The morphological differences highlight how precursor concentration influences coating quality, promoting either cohesive films (at high concentration) or defect-prone structures (at low concentration).

This comparison underscores the importance of optimizing precursor concentration in electrodeposition processes, as it directly impacts film morphology, stress distribution, and potential performance in protective or functional applications.

## 4. Conclusions

Based on the Design of Experiments (DoE), it is evident that the experimental conditions, particularly precursor concentration and current density, have a considerable impact on the performance of the electrodeposition–sintering process for YSZ ceramic coatings.

The consistent observation that S < R in all samples indicates material loss during the sintering stage, likely caused by volatilization of components, thermal contraction, or chemical reactions. Statistical analysis confirms that R is more variable than S, and both are significantly influenced by the selected process parameters.

The developed regression models for R and S show high predictive accuracy (R^2^ = 0.92 and 0.89, respectively). In the case of R, temperature showed limited statistical significance, suggesting it could be excluded to simplify the model. For S, all variables were relevant and retained.

Optical microscopy revealed that coatings obtained under central conditions are more uniform and homogeneous, while extreme conditions (e.g., high current density or concentration) led to thicker but structurally compromised coatings due to cracks and inhomogeneities. Thus, optimal performance requires balancing deposition yield with coating quality.

The optimal parameters identified through model prediction and experimental validation were as follows: 42.9 g·L^−1^ precursor concentration, 1.5 A·cm^2^ current density, 20 min of deposition, and 500 °C sintering temperature. Under these conditions, coatings exhibited minimal porosity and good morphological integrity.

While this study focused on surface morphology, deposition yield, and predictive modeling, future work will incorporate cross-sectional SEM/EDS analysis, XRD characterization of crystalline phases, and standardized adhesion testing to provide a more comprehensive evaluation of coating performance.

## Figures and Tables

**Table 1:** Chemical composition of the AISI 310 (P) stainless steel substrate [25].

Substrate	Composition (% Weight)	Geometry	
		S (mm^2^)	Thickness (mm)
P	0.25% C	30 × 20	3
	1.50% Si		
	2000 Mn		
	24.00% Cr		
	12.00% Ni		

**Table 2:** Tableting parameters.

Sample	Warm-Up Time (min)	Cooling Time (min)	Pressure (Bar)	Temperature (°C)	Mold Size (mm)
P	10	10	80	180	50

**Table 3:** Roughness measurement data [25].

Samples	Average Ra (μm)	Average Rq (μm)	Average Rz (μm)
P	0.106	0.160	0.930
PM	0.090	0.109	0.389
PMOx	0.098	0.124	0.477

**Table 4:** Variables, central value, maximum, minimum, and step.

Variable	Central Value	Step	Maximum	Minimum
Concentration (g/L)	42.9	17.55	78	7.8
Temperature (°C)	500	150	800	200
Current density (A/cm^2^)	1.5	0.75	3	0
Time (min)	20	9	38	2

**Table 5:** Coded essays.

Sample	Concentration	Temperature (°C)	Current Density (A/cm^2^)	Time (min)
1	0	0	0	0
2	1	1	1	1
3	0	0	0	0
4	0	0	0	0
5	0	0	0	0
6	0	0	0	0
7	−1	1	−1	−1
8	−1	1	1	−1
9	0	0	0	−2
10	0	0	0	0
11	−1	1	1	1
12	−1	1	−1	−1
13	0	0	0	2
14	0	−2	0	0
15	2	0	0	0
16	1	1	−1	−1
17	−2	0	0	0
18	−1	−1	−1	−1
19	1	−1	−1	1
20	0	0	2	0
21	1	−1	−1	1
22	1	−1	1	1
23	1	−1	1	−1
24	0	0	1	0
25	0	0	0	0
26	−2	0	−2	0
27	0	0	0	0
28	0	0	0	0
29	1	−1	1	1
30	0	−1	0	1
31	−1	−1	0	1
32	1	2	1	1
33	1	1	0	−1
34	−1	−1	0	0
35	0	0	1	0
36	0	1	1	−1

**Table 6:** Decoded essays.

Sample	Concentration	Temperature (°C)	Current Density (A/cm^2^)	Time (min)
1	42.9	500	1.5	20
2 *	60.45	650	2.25	29
3	42.9	500	1.5	20
4	42.9	500	1.5	20
5	42.9	500	1.5	20
6	42.9	500	1.5	20
7	25.35	650	0.75	11
8	25.35	650	2.25	11
9	42.9	500	1.5	2
10	42.9	500	1.5	20
11	25.35	650	2.25	29
12	25.35	650	0.75	11
13	42.9	500	1.5	38
14	42.9	200	1.5	20
15	78	500	1.5	20
16	60.45	650	0.75	11
17	7.8	500	1.5	20
18	25.35	350	0.75	11
19	60.45	350	0.75	29
20	42.9	500	3	20
21	60.45	350	0.75	29
22	60.45	350	2.25	29
23	60.45	350	2.25	11
24	42.9	500	2.25	20
25	42.9	500	1.5	20
26	7.8	500	0	20
27	42.9	500	1.5	20
28	42.9	500	1.5	20
29	60.45	350	2.25	29
30	42.9	350	1.5	29
31	25.35	350	1.5	29
32	60.45	800	2.25	29
33	60.45	650	1.5	11
34	25.35	350	1.5	20
35	42.9	500	2.25	20
36	42.9	650	2.25	11

* Sample 2, whose conditions match a sample previously published by the authors, has been included in this study as a comparative reference to validate the reproducibility of the model.

**Table 7:** Electrodeposition and sintering process performance.

Sample	Electrodeposition Yield (R) (mg·cm^−2^)	Sintering Yield (S) (mg·cm^−2^)
1	3.89	3.30
2	6.80	5.36
3	3.86	3.29
4	3.90	3.35
5	3.88	3.30
6	3.91	3.35
7	1.65	1,29.
8	3.25	2.57
9	3.87	3.29
10	3.85	3.28
11	4.08	3.12
12	1.68	1.30
13	3.90	3.34
14	3.92	3.35
15	5.97	4.68
16	2.25	2.09
17	1.25	1.03
18	1.64	1.45
19	2.52	2.25
20	6.90	5.79
21	2.52	2.27
22	6.03	5.43
23	5.60	5.10
24	4.01	3.42
25	3.89	3.33
26	0.85	0.70
27	3.87	3.29
28	3.89	3.29
29	6.05	5.44
30	4.02	3.60
31	3.66	2.70
32	6.02	4.30
33	3.75	3.41
34	2.65	2.39
35	4.01	3.41
36	3.29	2.64

**Table 8:** Summary of descriptive statistics for samples R and S.

Statistics	R (Electrodeposition)	S (Sintering)
Mean (average)	3.78	3.24
Median	3.89	3.30
Mode	3.89	3.29
Standard deviation	1.34	1.23
Minimum	0.85	0.70
Maximum	6.90	5.79
Range	6.05	5.09

**Table 9:** Regression equations for R and S and coefficients of determination (R^2^).

Dependent Variable	Regression Equation	R^2^
R	R = −0.45 + 0.08⋅C + 0.002⋅T + 1.20⋅DC + 0.03⋅t	0.92
S	S = −0.35 + 0.06⋅C + 0.003⋅T + 1.05⋅DC + 0.02⋅t	0.89

**Table 10:** *p*-values in the R and S regression models.

Regression Model	Constant Term	C	T	DC	t
R	*p* < 0.05	*p* < 0.05	*p* > 0.05	*p* < 0.05	*p* < 0.05
S	*p* < 0.05	*p* < 0.05	*p* < 0.05	*p* < 0.05	*p* < 0.05

## Data Availability

The authors confirm that the data supporting the findings of this study are available within the article.
